# Highly Pathogenic Avian Influenza Virus A/H5N1 Infection in Vaccinated Meat Duck Flocks in the Mekong Delta of Vietnam

**DOI:** 10.1111/tbed.12470

**Published:** 2016-01-08

**Authors:** N. V. Cuong, V. N. T. Truc, N. T. Nhung, T. T. Thanh, T. T. B. Chieu, T. Q. Hieu, N. T. Men, H. H. Mai, H. T. Chi, M. F. Boni, H. R. van Doorn, G. E. Thwaites, J. J. Carrique‐Mas, N. T. Hoa

**Affiliations:** ^1^ Oxford University Clinical Research UnitWellcome Trust Major Overseas ProgrammeHospital for Tropical DiseaseHo Chi Minh CityVietnam; ^2^ University of ScienceHo Chi Minh CityVietnam; ^3^ Sub‐Department of Animal HealthTien Giang ProvinceVietnam; ^4^ Centre for Tropical MedicineNuffield Department of MedicineUniversity of OxfordUK

**Keywords:** one health, zoonoses, vaccine, veterinary epidemiology, avian influenza virus, duck, H5N1

## Abstract

We investigated episodes of suspected highly pathogenic avian influenza (HPAI)‐like illness among 12 meat duck flocks in two districts in Tien Giang province (Mekong Delta, Vietnam) in November 2013. In total, duck samples from 8 of 12 farms tested positive for HPAI virus subtype A/haemagglutinin 5 and neuraminidase 1 (H5N1) by real‐time RT‐PCR. Sequencing results confirmed clade of 2.3.2.1.c as the cause of the outbreaks. Most (7/8) laboratory‐confirmed positive flocks had been vaccinated with inactivated HPAI H5N1 clade 2.3.4 vaccines <6 days prior to onset of clinical signs. A review of vaccination data in relation to estimated production in the area suggested that vaccination efforts were biased towards larger flocks and that vaccination coverage was low [21.2% ducks vaccinated with two shots (range by district 7.4–34.9%)]. The low‐coverage data, the experimental evidence of lack of cross‐protection conferred by the currently used vaccines based on clade 2.3.4 together with the short lifespan of meat duck flocks (60–70 days), suggest that vaccination is not likely to be effective as a tool for control of H5N1 infection in meat duck flocks in the area.

## Introduction

Highly pathogenic avian influenza (HPAI) virus subtype A/H5N1 (haemagglutinin 5 and neuraminidase 1) remains endemic in poultry populations in areas of Vietnam and other South–East Asian countries, in spite of large investment of resources on control since 2003, when the virus emerged in the region (Pfeiffer et al., 2013). In the Mekong Delta region of southern Vietnam, HPAI H5N1 outbreaks have continued to be reported in poultry although in much lower numbers compared with the initial period 2003–2005. For example, during the 2‐year period October 2012–September 2014, 13 confirmed HPAI H5N1 outbreaks were reported from 8 of 13 provinces of the Mekong Delta region (FAO, 2014). In contrast to this low frequency of disease outbreaks, HPAI H5N1 viruses are consistently detected in poultry flocks in the Mekong Delta and other regions of Vietnam (Phan et al., 2013; Nguyen et al., 2014a).

Ducks are very important as a reservoir species for HPAI H5N1 viruses, as infected ducks often remain asymptomatic (Kim et al., 2009; Cha et al., 2013). In the Mekong Delta of Vietnam, duck farming systems typically involve free‐range rice grazing, a practice that has been associated with transmission of HPAI H5N1 infection (Minh et al., 2010; Henning et al., 2013).

HPAI H5N1 viruses have been grouped into different clades, based on the phylogenetic characterization of their haemagglutinin gene (WHO/OIE & FAO H5N1 Evolution Working Group, 2014). In the Mekong Delta region, variants of clade 1 (and its subvariants 1.1, and 1.1.1 and 1.1.2) were identified in 2003 and were predominant until 2012. Late in 2012, clade 2.3.2.1 was introduced and co‐circulated alongside clade 1 in southern Vietnam (Le and Nguyen, 2014).

In Vietnam and other countries such as China, Egypt and Indonesia, vaccination of poultry remains an important tool to control HPAI H5N1 infection (Swayne, 2012). In Vietnam, authorities have been sponsoring vaccination campaigns aiming at controlling HPAI H5N1 infection in poultry in areas considered to be at high risk, including the Mekong Delta provinces since September 2005 (FAO, 2011). The most common HPAI H5N1 vaccines used in Vietnam are inactivated preparations including clades 0 (‘Re‐1’, manufactured by Zhaoqing Dahuanong, China, and ‘Vifluvac’, manufactured by Navetco, Vietnam), 2.3.4 and 2.3.2.1 (‘Re‐5’ and ‘Re‐6’, respectively, both from Zhaoqing Dahuanong) matching the circulating viruses. The recommended vaccination regime for young ducks to achieve full protection consists of one shot at 14 days of age followed by a booster shot 28 days after Cha et al. (2013). Vaccines are provided free of charge by the government authorities to owners of chicken and duck farms with <2000 birds. Vaccination is typically performed by commune animal health workers.

### The outbreaks

In March 2013, the Sub‐Department of Animal Health of Tien Giang (SDAH‐TG) was alerted of suspected cases of HPAI H5N1 in mixed poultry farms in the province. Through October 2013, outbreaks were suspected in 43 farms belonging to 7 districts, 10 of which were confirmed as H5N1 positive by RT‐PCR. During the month of November 2013, suspect outbreaks continued to be reported in the districts of Cho Gao, Tan Phu Dong and Go Cong Dong. Here, we report on the investigation of 12 meat duck farms with suspected HPAI‐like illness in Cho Gao and Go Cong Dong districts during this period. Initial enquiries indicated that several duck flocks affected had been vaccinated immediately before onset of clinical signs. A full comprehensive investigation of these outbreaks was carried out. Such full investigations are uncommon in the area for logistic and economic reasons. The aims of the study were as follows: (i) to describe the outbreaks and to identify and characterize the causative agent (HPAI H5N1 and clade) in order to determine whether vaccination strains were involved; (ii) to characterize the genetic diversity and possible zoonotic risks associated with the HPAI H5N1 outbreak strains; and (iii) to review the vaccination strategy in the area and determine its potential effectiveness as a control tool for HPAI in the Mekong Delta region of Vietnam.

## Materials and Methods

### Farm visits and samples

Visits to farms with duck flocks showing symptoms compatible with HPAI H5N1 were conducted as soon as it was practicable following notification by the farmers. During the farm visits, veterinarians collected information about the episode of disease (clinical signs, mortality, morbidity) as well as demographic and vaccination data using standardized forms. In each farm, a diagnostic necropsy was conducted, involving the collection of lung and spleen tissues from three ducks (i.e. 6 specimens per farm) in aseptic conditions. In addition, from each farm 5 to 10 environmental samples were collected, including different combinations of the following: feathers from the pen floor, floor swabs (approximately 1 square metre) and drinker/pond sediment. All visits to duck farms were conducted by veterinarians affiliated to the Sub‐Department of Animal Health in Tien Giang (SDAH‐TG) and the District Veterinary Stations (DVS) of Cho Gao and Go Cong Dong districts.

### Laboratory analyses

Lung and spleen tissues samples were homogenized by TissueLyser machine (Qiagen) using silica bead. All other samples (environmental and feather samples) were vortexed well for 2 min, followed by 5‐min centrifugation at 9300 *g*. RNA was extracted from the 200 *μ*l processed supernatant samples using Viral RNA MiniKit (Qiagen, Hilden, Germany). Viral RNA extracts were screened for Influenza virus A and HPAI H5N1 by real‐time RT‐PCR using the US‐CDC protocol (US‐CDC, 2007). Samples testing positive for HPAI H5N1 were confirmed by sequencing the HA and NA genes after performing conventional RT‐PCR. Full HA sequence was used for clade typing. The primer for PCR and sequencing the HA gene were as follows: fragment A ~560 bp [Ha4‐F2: AGCAGGGGTTCAATCTGTCAAAA3 (Hoper et al., 2009) and HE‐540R: GCTCCTCTTTATTGTTGGG3 (Guan et al., 2002)], fragment B ~800 bp [HA5‐inner‐F: AATGACCTCTGTTACCCAGG and HA5‐inner‐R: GGTACCCATACCAACCATCT, (both self‐designed)], fragment C ~750 bp [H5CDCb‐F: GGAATGCCCCAAATATGTGAAATCAA (US‐CDC, 2007) and Ha4‐R2 AGTAGAAACAAGGGTGTTTTTAACTA (Hoper et al., 2009)]. The primers for PCR and sequencing of the NA gene were as follows: fragment A ~800 bp [NA_F1: 5′‐CTATAGGGAGCAAAAGCAGGAGT‐3′, NA_R1: 5′‐TAACAGGARCAYTCCTCRTARTG‐3′] and fragment B ~600 bp [NA_F2: 5′‐CAYTAYGAGGARTGYTCCTGTTA‐3′ and NA_R2: 5′‐CACTATAGAAGTAGAAACAAGGAGTTTTTT‐3′] (Ryabinin et al., 2011).

Alignments were performed with ContigExpress (Vector NTI Suite 7) and maximum‐likelihood phylogenetic inference was performed with RAxML using 100 bootstrap replicates (Stamatakis et al., 2008) and a GTR+Γ model of nucleotide substitution. Several important mutations associated with virulence in host, or associated with stability and conformation of HA protein, were investigated by comparing with the published data. All HA sequences we deposited into GenBank with accession numbers KR905399–KR905418.

### Data on duck production and HPAI H5N1 vaccination coverage

Information on types of vaccine and number of ducks HPAI H5N1 vaccinated with one and two shots in Tien Giang province was available for each district on 2‐monthly periods for the year 2013. The vaccination coverage for duck flocks was estimated from the official annual farm census and from vaccine administration data. In the Mekong Delta region of Vietnam, duck flocks are typically raised as single age. Depending on the breed and feeding system, the lifespan of a duck production cycle is 2–3 months and 1–2 years for meat and layer/breeding flocks, respectively. Meat ducks represent ~75% of the total census (Men, 2010), and their production cycle is typically synchronized with the rice production cycle (2–3 crops per year). The annual duck production in the province (by district) was estimated by multiplying the number of meat ducks (census data) by a factor of 2.5, and the number of layer/breeding ducks by 1, assuming a 3 : 1 ratio for meat and layer/breeding flocks. The census data (number of ducks by district) were available for two categories of duck farms, <200 and 200–2000. Owners of flocks with >2000 ducks need to source the HPAI H5N1 vaccine from the private market; therefore, the coverage for ducks raised in such farms could not be estimated. No specific data on the additional booster vaccination required for layer/breeding flocks were available, and therefore, the calculations were made assuming all ducks require two vaccination shots, regardless of production purpose. Records on confirmed and unconfirmed HPAI H5N1 outbreaks in duck flocks during 2013 in the province were available. All data were provided by SDAH‐TG.

### Statistical methods

Comparisons of proportions were performed using chi‐square tests and Fishers’ exact test when appropriate. Comparisons of vaccine coverage (expressed as number of ducks vaccinated with one and two shots divided by the estimated population) and the number of outbreaks (duck farms, farms without ducks) by time period, district and type of flock (<200, 200–2000 ducks) were carried out using standard binomial tests. To investigate the association between overall vaccine coverage, time period and observed number of outbreaks (outcome), a hierarchical multivariable Poisson model was built with the variable district as a random (cluster) variable and the variables ‘time period’ and ‘number of ducks vaccinated’ (log) as independent variables. In each model, the number of estimated ducks produced per month was coded as an offset variable. Analyses were performed using the lme4 package with R statistical software (www.r-project.org).

## Results and Discussion

### Outbreak descriptive data

A total of 12 farms (9 located in Cho Gao and 3 in Go Cong Dong) with duck flocks suspected to be infected with HPAI H5N1 were investigated 2–12 days after onset of clinical signs. Before onset of clinical signs, flocks had a median of 1450 [inter‐quartile range (IQR): 725–1575] ducks each. The median age of flocks at onset of symptoms was 28 days [IQR 25.8–30.0]. All farmers raised their ducks as single‐age flocks. The median morbidity was 41.9% [IQR 21.3–55.6%], and the median mortality was 16.0% [IQR 3.0–42.1%]. All (10/10) farms with clinical sign reports reported central nervous system disorders (circling, ataxia, etc.). Nine flocks (75%) had recently been HPAI H5N1‐vaccinated a median of 3 days [IQR 2–5] prior to onset of clinical signs (Table 1). The reported mortality (16.0%) was lower compared with that observed in 2.3.2.1 clade outbreaks in India, Bangladesh and Indonesia (>60%) (Nagarajan et al., 2012; Dharmayanti et al., 2014; Haider et al., 2015). In our study farms, the variable mortality was statistically correlated with the length of time between onset of signs and farm visit (Pearson's correlation = 0.61, *P* = 0.01) (data not shown). In the study by Haider et al. (2015) (Bangladesh), mortality in ducks due to HPAI H5N1 clade 2.3.2.1a was 47% after 14 days of onset of disease, whereas in our study farms were visited and data collected within a median of 4 days of disease onset [IQR 3–4].

**Table 1:** Descriptive data and H5N1 test results in 12 duck flocks in two districts of Tien Giang province

Farm ID	District	Date of onset	No. ducks	Flock age (days)	Morbidity (%)	Mortality (%)	Day of vaccination	Days from vaccination to onset	Days from onset to sampling	H5N1 test results (No. Pos./Total)		Clinical signs
										Organ tissues	Environmental samples	
T03	CG	8‐Nov	1200	25	41.7	16.7	NV	–	12	6/6	NT	CNS disorders, cloudy eyes
T02	CG	11‐Nov	1500	26	73.3	70.0	6‐Nov	5	9	0/6	0/7	CNS disorders, cloudy eyes
T01	CG	16‐Nov	500	30	50.0	14.0	13‐Nov	3	4	5/6	2/8	CNS disorders, cloudy eyes
T05	CG	18‐Nov	1500	20	21.3	8.0	16‐Nov	2	4	5/6	0/10	CNS disorders, cloudy eyes
T06	GC	18‐Nov	1900	30	42.1	42.1	14‐Nov	4	4	0/6	NT	White stools, CNS disorders
T07	GC	18‐Nov	1800	28	2.8	0.8	NV	–	4	0/6	NT	CNS disorders, cloudy eyes
T08	GC	19‐Nov	1500	67	13.3	1.3	NV	–	3	0/6	NT	NA
T04	CG	19‐Nov	500	26	30.0	3.0	13‐Nov	6	3	6/6	0/10	NA
T09	CG	21‐Nov	1,950	30	25.6	15.4	21‐Nov	0	3	6/6	0/8	CNS disorders, cloudy eyes
T13	CG	26‐Nov	800	50	81.3	75.0	25‐Nov	1	5	4/6	2/10	CNS disorders, cloudy eyes
T12	CG	28‐Nov	1400	28	50.0	28.6	25‐Nov	3	3	6/6	2/10	CNS disorders, cloudy eyes
T14	CG	29‐Nov	450	25	55.6	22.2	24‐Nov	5	2	6/6	0/6	CNS disorders, cloudy eyes

CG, Cho Gao district (Tien Giang province); GCD, Go Cong Dong district; Pos., positive; NV, not vaccinated; NT, not tested; NA, not available; CNS, central nervous system.

### Real‐time RT‐PCR testing results

Eight of the 12 (66.6%) duck farms investigated, all in Cho Gao district, tested positive for HPAI H5N1 by real‐time RT‐PCR. All lung (24/24) samples, 20 of 24 (83.3%) spleen samples and 6 of 62 (9.7%) environmental samples from the eight farms also tested positive for HPAI H5N1 by real‐time RT‐PCR. Positive environmental samples included 4 feather samples (from 2 farms), one floor swab sample and one drinker sediments sample. Cycle threshold (CT) values ranged from 25.8 to 30.0 (tissue samples) and 25.5 to 31.4 (environmental samples). Seven of the 8 laboratory‐confirmed flocks had been vaccinated against HPAI H5N1, versus 2 of the 4 negative flocks (Fisher's exact test; *P* = 0.129) (Table 1).

### Sequencing data

**Figure 1:**
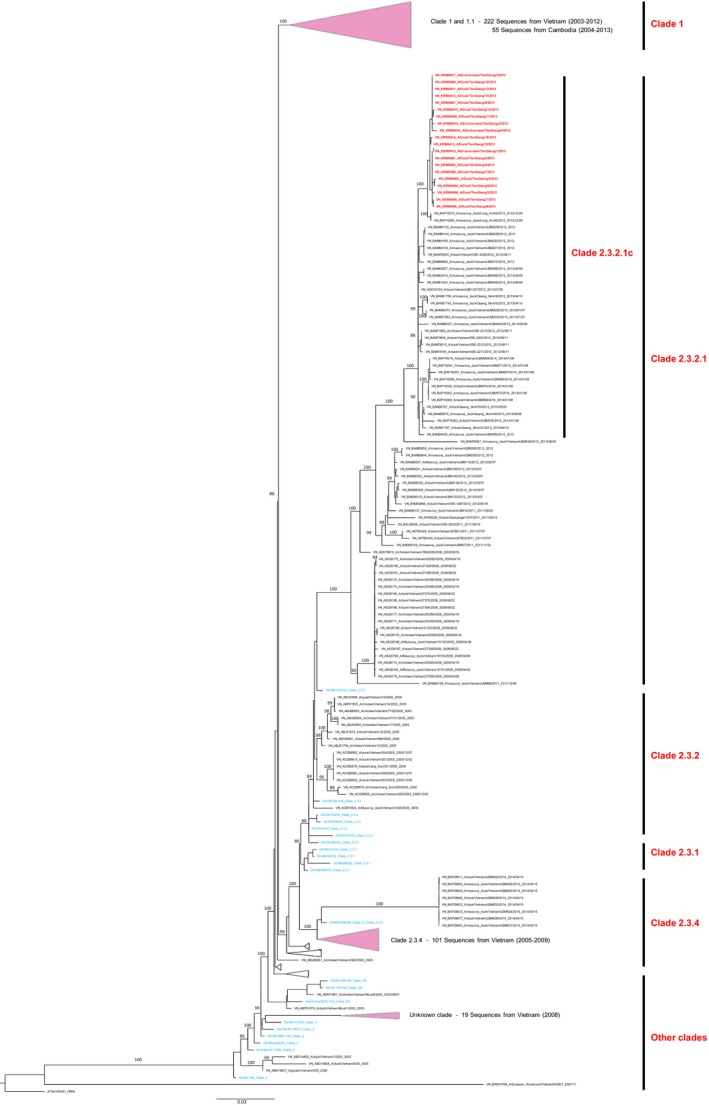
Maximum‐likelihood phylogenetic tree of influenza A subtype H5 HA segment (full length) for viruses isolated in Vietnam and Cambodia. Bootstrap values ≥80 are shown on key nodes. The majority of H5 sequences in GenBank from Vietnam and Cambodia are Clade 1 sequences, and these are shown in collapsed form at the top of the tree. More than 100 sequences are from Clade 2.3.4; these are shown collapsed in the bottom of the tree except for eight sequences samples in April 2014, which are shown explicitly. Sequences shown in blue are WHO clade markers, and the major clade shown in the tree is Clade 2.3.2, which includes viruses circulating from 2004 until the present. The 20 sequences shown in red at the top of the clade 2.3.2.1c branch correspond to the viruses described in this study. All HA sequences we deposited into GenBank with accession numbers KR905399–KR905418.

The products with the lowest CT values by real‐time RT‐PCR values from each of the 8 laboratory‐confirmed positive farms were used to sequence the *HA* gene using direct sequencing as previously described (Guan et al., 2002). A total of 20 products were investigated, two from lung tissue specimens from each of the farms, and four environmental samples. Sequencing results indicated that HPAI H5N1 clade 2.3.2.1c was present in all samples (Fig. 1). All sequences contained important mutations associated with virulence in humans, including those responsible for affinity for the human α2,6‐sialic acid receptor (S224G, A113, K189R, K112E) (Shore et al., 2013) and the multibasic cleavage site of HPAI HA (SPQGERRRKKR | G), (Chutinimitkul et al., 2010), as well as substitutions associated with stability of trimer structure of the HA protein (P217S) (Shore et al., 2013). All 20 HA gene sequences analysed were phylogenetically related to clade 2.3.2.1.c, clustered in a larger group containing sequences of viruses that were isolated from poultry in other regions of Vietnam during 2012–2014. Since 2011, clade 2.3.2.1 has been replacing the previously dominating clade 2.3.4 in northern and central Vietnam. This clade subsequently evolved into its variants (2.3.2.1a/b/c), and became widespread throughout the country (WHO, 2012). This 2.3.2.1c clade was believed to have emerged in the Mekong Delta region at the end of 2013.

Since 2012, when clade 2.3.2.1c was first identified in Vietnam, only one (fatal) human case has been confirmed as infected with this clade (in January 2014) in the Mekong Delta province of Dong Thap, adjacent to Tien Giang province, where this study was conducted (WHO, 2014; Thor et al., 2015). In Vietnam, a total of 127 HPAI H5N1 human cases have been confirmed from 2003 to 2014 (WHO, 2015), and for 87 human cases, clade information is available. The most common clades in human patients were clade 1 (55 cases; circulating in poultry for 6 years, from 2003 to 2009), followed by clade 2.3.4.3 (14 cases; circulating in poultry for 4 years, from 2007 to 2010), clade 1.1.1 (5 cases, circulating for 6 years, from 2009 to 2014), 2.3.4.1 (4 cases, circulating in poultry for 3 years, 2007 to 2009), 2.3.4.2 (3 cases, circulating for 3 years, 2008 to 2010), and 1.1.2 (3 cases, circulating for 6 years, 2009–2014). For each of the other clades, there have been two or fewer cases reported. The confirmation of one human case with 2.3.2.1c over 3 years’ circulation of this clade in poultry confirms its zoonotic potential, although it is probably low. Outside Vietnam, another case attributed to clade 2.3.2.1c was reported in Canada resident who infected shortly after returning from China in December 2013 (Pabbaraju et al., 2014).

### Duck production, HPAI H5N1 vaccination and outbreak data

Annual duck production in Tien Giang was estimated in ~3.8 million ducks. In 2013, a total of 38 suspected outbreaks were reported in duck flocks in the province, 20 (52.6%) of which were molecular confirmed as HPAI H5N1. In addition, 22 outbreaks were investigated in farms without ducks (including quails, chickens and Muscovy ducks), of which 15 (65.2%) were confirmed. Among confirmed duck outbreaks, 16 were in farms with 200–2000 ducks, and 4 in farms with <200 ducks. The *per capita* IRR (incident risk ratio) for ducks in the larger farm category was 4.03 [95% CI = 1.35–12.07], *P* = 0.013) (Table 2). Overall vaccination coverage was estimated at 64.3% for one shot (range 24.6–90.3% by district) and 21.2% for two shots (range 7.4–34.9%). Vaccination coverage with two shots for ducks in larger farms (200–2000 ducks) was significantly higher compared for ducks in smaller farms (<200 ducks) (31.8% versus 2.8%, respectively) (*χ* ^2^ *P* < 0.001). Vaccination data for the other key species (chickens) suggest an even lower intensity of vaccination (about 4.9 million doses for a census population of 5.8 million) (data not shown). After adjusting for district, vaccination coverage of ducks with one shot was highest in November–December (62.2%) and lowest in January–February (35.1%). Vaccination coverage for two shots was greatest in December (31.0%) and lowest in January–February (5.3%). Vaccination coverage with one shot was greatest for My Tho and Cho Gao (90% and 85.4%) and lowest for Go Cong and Cai Be (35.9% and 24.6%, respectively). These former two districts had the highest coverage of ducks vaccinated with two shots (32.0–34.1%), whereas Go Cong and Cai Be had the lowest (10.9% and 7.4%, respectively). After adjusting for district, vaccination coverage of ducks was highest in November–December (62.2% ducks vaccinated with one shot; 31.0% ducks with two shots) and lowest in January–February (35.1% ducks with one shot, 5.3% two shots). Vaccination coverage for ducks with one shot was greatest for My Tho and Cho Gao (90% and 85.4% ducks with one shot) and lowest for Go Cong and Cai Be (35.9% and 24.6%, respectively). My Tho and Cho Gao had the highest coverage of ducks vaccinated with two shots (34.1% and 32.0%), whereas Cai Be and Go Cong had the lowest (7.4% and 10.9%, respectively). Univariable random effects models indicated that both the number of ducks vaccinated with one shot and number of ducks vaccinated with two shots and the time period were associated with the number of confirmed outbreaks (*P* < 0.05). However, when the three variables were modelled together, only the number of ducks vaccinated with one shot remained significant (IRR = 4.12; SE = 1.12) (log) (*P* < 0.001), but not the number of ducks vaccinated with two shots (IRR = 0.416; SE = 0.30 (*P* = 0.16), or the time period (data not shown).

**Table 2:** Estimated vaccination coverage in the province of Tien Giang by district and farm size in relation to duck census data (2013)

	Duck census data	Estimated annual duck production	Vaccination data				No. duck flocks confirmed with H5N1
			No. ducks receiving one shot	Coverage (%)	No. ducks receiving two shots	Coverage (%)	
By district^a^							
Cai Be	417 207	886 563	218 523	24.6	65 218	7.4	1
Cai Lay	257 524	547 239	255 935	46.8	118 811	21.7	2
Tan Phuoc	71 343	151 604	94 914	62.6	36 910	24.3	1
Chau Thanh	122 283	259 849	190 953	73.5	50 643	19.5	4
My Tho	25 111	53 361	48 166	90.3	18 600	34.9	0
Cho Gao	144 739	307 570	262 845	85.5	99,643	32.4	9
Go Cong Tay	316 687	672 960	549 294	81.6	120 104	17.8	0
Go Cong	105 841	224 912	80 795	35.9	24 565	10.9	0
Go Cong Dong	159 675	339 309	225 780	66.5	45 188	13.3	0
Tan Phu Dong	51,671	109 799	83 423	76.0	32 895	30.0	3
Total	1 672 079	3 553 166	2 010 628	64.3	612 577	21.2	20
By farm size							
<200 ducks	839 559	1 784 061	378 380	21.2	49 121	2.8	4
200–2000 ducks	832 520	1 769 105	1 632 248	92.3	563 447	31.8	16
>2000 ducks	210 200	446 675	NA	NA	NA	NA	NA
Total	1 882 279	3 999 841	NA	NA	NA	NA	20

a Data for farms with up to 2000 ducks. NA, No data available.

The analysis of vaccine coverage data suggests that vaccination efforts are biased towards farms with a perceived (current or historical) higher risk of HPAI H5N1, either because of their size (larger flocks), their location (i.e. districts or areas close to confirmed or unconfirmed outbreaks), or the time of the year (i.e. when outbreaks are reported).

The Mekong Delta region is considered endemic for HPAI H5N1 circulation, with high levels of detection of infection in poultry, often without clinical signs (Nguyen et al., 2014b).

The estimated vaccine coverage in duck flocks, although low (21.2% ducks receiving two shots), was similar to that reported in previous studies (22–26%) (Hinrichs, 2010). Even in the hypothetical case that protective vaccine strains had been used, vaccination efforts are unlikely to be sufficient in preventing transmission of HPAI H5N1, given that the required herd immunity levels to halt transmission need to be in excess of 50% (Pantin‐Jackwood and Suarez, 2013). The administration of two vaccine doses to duck flocks within the required schedule presents considerable logistic challenges (Henning et al., 2011). Our data indicate that vaccines were rarely administered at the correct timing, as half of the duck population received their first shot after 35 days old (data not shown). Improperly implemented vaccination programmes may play a role in the emergence of antigenic drift in currently circulating high‐ and low‐pathogenicity avian influenza viruses (Tosh et al., 2014; Beato et al., 2014; Escorcia et al., 2008). This poses an additional challenge to countries that rely on mass vaccination control programmes to control HPAI H5N1, such as China, Indonesia, Egypt and Vietnam. Over a 10‐year period, the reported vaccination coverage in these countries ranged from 14% (China) to 69.9% (Egypt) (Swayne, 2012). Co‐circulation multiple clades represents an additional challenge to the design of mass vaccination programmes.

Our study highlights the difficulty in estimating vaccination coverage from census data given the seasonality of duck production in the Mekong Delta. An accurate assessment of vaccination coverage should take into account seasonal variations in duck populations. In our study, we cannot conclusively determine whereas the increased vaccination efforts during November and December reflect genuine increases in duck populations, a perceived higher risk of infection or both. This is the season with a highest production to meet the demand for protein due to the incoming Tet festival in January/February.

In summary, we have documented a number of outbreaks in ducks associated with the introduction of HPAI H5N1 virus clade 2.3.2.1c into the Mekong Delta province of Tien Giang during 2013. The identified virus had important mutations associated with virulence in humans, and the identification of a human fatal case confirms its zoonotic transmission and virulence capacity. The 2.3.4 clade‐based vaccine formulations used in the area at the time are not effective against this emerging new clade (Le and Nguyen, 2014). Our observations suggest that even in the case of effective vaccines being used, the current vaccination efforts are clearly insufficient to protect duck flocks in the area. As an alternative to mass vaccination of meat duck flocks, we recommend focusing on better vaccination of layer and breeding flocks, improving biosecurity and limiting flock movements into and out of high‐risk areas.

## Author Disclosure Statement

No competing financial interests exist. MFB has worked as a paid consultant to Visterra, Inc. in Cambridge, MA, USA.
